# Laser-induced changes in the gene expression, growth and development of *Gladiolus grandiflorus* cv. “White Prosperity”

**DOI:** 10.1038/s41598-024-56430-6

**Published:** 2024-03-15

**Authors:** Manar Hassan, Shimaa A. Shaaban, Rasha A. El Ziat, Khaled A. Khaled

**Affiliations:** 1 National Institute of Laser Enhanced Sciences (NILES), Department of Laser Application in Metrology, Photochemistry and Agriculture (LAMPA,), Cairo University, PO 12613, Giza, Egypt; 2 Faculty of Agriculture, Department of Agricultural Botany, Cairo University, PO 12613, Giza, 12613 Egypt; 3 Faculty of Agriculture, Department of Ornamental Horticulture, Cairo University, PO 12613, Giza, Egypt; 4 Faculty of Agriculture, Department of Genetics, Beni-Suef University, PO box 62517, Beni Suef, Egypt

**Keywords:** *Gladiolus grandifloras*, He–Ne, Anatomical structures, SSR marker, Genetics of gladiolus, Biological techniques, Biotechnology, Genetics, Plant sciences

## Abstract

Corms of *Gladiolus grandiflorus* cv. “White Prosperity” was irradiated via red laser at wavelength 635 nm. Various morphological, flowering, elemental and chemical characterizations were studied. Irradiation with different power (5, 20, and 50 mW) and various irradiation time (0.0, 0.5, 1, 3, 5 and 10 min) was studied. Several characters), totaletermined include vegetative growth parameter (spouting days, plant height (cm), leaves number, leaves fresh and dry weights (g/plant), diameter of plant middle part (mm) and leaf area (cm^2^), floral parameters (flowering days, vase life (day), fresh and dry weights of inflorescence (g/plant), number of flowers per inflorescence, inflorescence length(cm), flowers diameter(cm), number of corms per plant, corms fresh weight(g/plant), circumference/ corms), pigments [total chlorophylls in leaves (SPAD), anthocyanin content (mg/100 g F.W.) in petals], NPK (%) in new corms and chemical composition in corms; total carbohydrates (%),total phenol (μg CE/g (%),total flavonoid (μg CE/g) (%), antioxidant (DPPH IC50 (μg /ml (%), and proline content (μ moles/g). The results showed that the medium level (20 mW) of He–Ne laser at 5 min caused favorable changes in the leaf anatomical structures and other studied characters followed by the low level (5 mW) of He–Ne laser at 5min. 112 bands emerged from 22 SSR primers, ranging between 130 and 540 bp, with 32 bands having polymorphism ranging from 17–100%. Out of the 22 SSR primers, 3 primers exhibited a high polymorphism percentage, i.e., SSR6, SSR16 and SSR22 which exhibited 7 positive markers. These findings revealed the efficiency of SSR primers for differentiating gladiolus plants and revealed that some alleles were affected by laser in their corms and the expression resulted in color or abnormalities in leaves and/or flowers. Mutation in some alleles could result in abnormalities like mutation in the allele with 410 bp revealed by SSR16.

## Introduction

Gladiolus is derived from the native plants of South and Central Africa, as well as the Mediterranean region. It belongs to the family Iridaceae. Gladiolus is an economic flowering bulb plant used as a landscape plant in home gardens and in decoration as a lovely and rich-colored cut flower spike with a relatively long vase life^1^.

Flowers create a motivating, pleasing, colorful and fragrant environment for people, also they are used to decorate houses, offices, and to complete ceremonies as purpose friendships, and marriage. Additionally, gladiolus is used for making perfumes, medicine, essential oil, and other cosmetics purposes^2^.

Laser is one of the most advanced and successful physical techniques used recently to bio-stimulate seeds and vegetative parts of plants. The term “LASER” is an acronym for “Light Amplification by Stimulated Emission of Radiation,” and it refers to a technology that produces coherent monochromatic light beams with specific optical characteristics, such as intensity, emission wavelength, beam divergence, these beams interact with plant cells, which absorb and store radiant energy^3^. The synergism between the polarized monochromatic laser beams and photoreceptors forms was the basis of the laser stimulation mechanism in plant's physiological development. Considerable studies support the bio-stimulating effect of laser on various plant tissues and organs. Plants utilize their photoreceptors to absorb light and regulate all phases of a plant's growth^4^**.** Radiation has been used for inducing mutations and examining the variability that can be obtained through mutation breeding, flowers are now brighter and deeper in hue^5^. Several beneficial effects, including stimulation of plant growth during all stages of vegetation, such as shorthand the time needed for germination and flowering, increase in the number of flowers per plant, qualitative and quantitative increase in yield, etc., were achieved by irradiating seeds and other plant parts^6,7^. Many factors affect the way laser radiation works, including the wavelength used, exposure period, power, dosage, and irradiation technique (constant or pulse)^8^. The primary effect of laser irradiation after absorption shows thermal and electromagnetic effects^9^.

Targeted genes related to various traits were genotyped, barcoded, and their gene expression is assessed using molecular markers^10–12^. To determine the gene expression and QTLs in plants, markers including amplified fragment length (AFLP), simple sequence repeats (SSR), and start codon targeted marker (SCoT) are utilized^7,13–17^. This method has advantages over all others since it is less expensive, more efficient, easier to use, quicker, and more easily reproducible^18^. One of the most popular molecular markers in plant breeding and genetic diversity are SSR markers, commonly known as microsatellites Due of their co-dominant inheritance, high repeatability, high polymorphism, and multi-allelic nature, SSRs are the ideal marker system^19^. The simplest and most affordable source for SSR production is chloroplast SSR (cpSSR), which are frequently employed to evaluate the genetic diversity of Gladiolus^12,20,21^. To achieve the needed genetic variability in a variety of ornamental plant species, irradiation with diverse types of radiation has been applied^7,17^. Commercially, ornamental plants are admired and in high demand because they have a wide range of floral colors and consistent shapes. In comparison to the control plant, a high number of mutations produced by irradiation technology assist in introducing new, improved variations^22,23^.

No recorded data are available on laser irradiation effect on *gladiolus.* Therefore, the aim of this research is to investigate the effect of He–Ne laser irradiation on *Gladiolus grandiflorus* cv. “White Prosperity” and response of growth, quality characteristics, genetic attributes, and anatomy of gladiolus when exposed to red laser light.

## Results

### Vegetative growth characters

Data in Tables 1 and 2 showed that treated gladiolus corms with three laser power levels (5, 20, and 50 mW) resulted significantly increase in the studied growth parameters, (plant height, number of leaves/plant, leaf area, fresh and dry weight of leaves (g)/plant) compared with control. The best results were obtained after irradiation with medium power at 20 mW for 5 min. This treatment gave significantly higher plants (117.2 cm) than the control plants 68.33 cm) in both seasons (Table 2). The best results for the number of leaves per plant (L/p) were recorded at 20 mW for 5 min irradiation (13 L/p) in comparison with the other treatments. Additionally, it was detected that, treating corms of Gladiolus plants with 20 mW for 5 min was more effective than other used combinations of powers and irradiation timing on fresh weight (46.13 g/plant) and dry weights (18.9 g/ plant) of leaves per plant in both seasons as compared to control. Also, the highest leaf area (107.450 cm^2^) and diameter of plant middle part (57.28 mm) were recorded in 20 mW for 5 min irradiation compared to control (Table 2). All previously mentioned growth characters increased with increasing both power and exposure times except high power (50 mW) irradiation with long irradiation time (10 min).

**Table 1:** Mean square for the effect of He–Ne Laser (L), irradiation time (T) and their interaction on vegetative growth parameters of (*Gladiolus grandiflorus* L.) cv.”White Prosperity”.

Traits	Source of variation			Error	Cv
	Treatment				
	He–Ne Laser(L)	irradiation time (T)	L × T		
Days to corms sprouting	229.34***	4.82***	10.08***	0.693	7.09
Plant height (cm)	2178.62***	285.38***	125.50***	2.932	1.973
N. of leaves/plant	33.48***	6.83***	1.44***	0.271	5.333
FW of leaves (g/plant)	1516.04***	106.72***	25.03***	0.669	2.411
DW of leaves (g/plant)	152.89***	13.37***	3.25***	0.173	3.270
Diameter of middle part of plant (mm)	407.78***	12.77***	9.42***	0.834	1.803
Leaf area (cm2)	4904.14***	219.10***	135.08***	1.520	1.517
Days to flowering	1099.72***	69.96***	97.53***	2.600	1.82
No.floLets/spike	15.533***	9.000***	2.714***	0.171	4.43
florlets dimater(cm)	72.66***	6.64***	4.306***	0.544	7.06
spike length (cm)	1901.54	105.71	38.89	4.671	5.35
FW of flower (g/ plant)	2777.63***	262.09***	118.1***	1.570	2.05
DW of flower (g/plant)	51.95***	34.18***	6.20***	0.608	4.91
Vase life (days)	28.533***	3.058***	2.27***	0.434	6.74
N. of corms/plant	435.76***	131.75***	67.05***	0.655	5.80
FW of corms (g/plant)	760.57***	582.02***	296.49***	1.785	3.73
Circumference/corms	131.42***	5.60***	5.25***	0.145	3.69

*, **, *** significant at *P* ≤ 0.05, *P* ≤ 0.01, *P* ≤ 0.001, respectively.

**Table 2:** Vegetative growth characteristics (*Gladiolus grandiflorus* L.) cv.”White Prosperity” affected by the interactions between He–Ne Laser mW and different irradiation time during mean of seasons 2022/ 2023.

He–Ne laser (L)	Irradiation time (min)	Days to spourting	Plant height (cm)	N. of leaves/plant	FW of leaves (g/plant)	DW of leaves (g/plant)
Control	0.5	16.00 ± 0.57 cd	68.33 ± 1.02 k	7.17 ± 0.33 h	17.75 ± 0.56 m	7.11 ± 0.15 m
	1	17.33 ± 0.33 bc	68.67 ± 0.17 jk	7.50 ± 0.29 h	19.48 ± 0.16 l	8.39 ± 0.56 l
	3	16.33 ± 0.17 cd	69.83 ± 0.45 jk	8.00 ± 0.50 gh	20.66 ± 0.26 kl	7.78 ± 0.09 lm
	5	19.17 ± 0.33 a	71.67 ± 0.62 j	7.67 ± 0.17 h	21.47 ± 0.20 k	9.11 ± 0.02 k
	10	18.67 ± 0.67 ab	71.67 ± 0.73 j	8.67 ± 0.17 fg	20.87 ± 0.15 k	9.10 ± 0.45 k
5 mW	0.5	15.33 ± 0.93 d	81.00 ± 0.58 i	8.67 ± 0.45 fg	24.58 ± 0.71 j	10.9 ± 0.20 j
	1	11.00 ± 0.76 e	87.67 ± 1.67 gh	10.5 ± 0.50 cd	34.27 ± 1.00 h	12.3 ± 0.32 hi
	3	9.000 ± 0.02 hij	97.33 ± 0.89 c	10.5 ± 0.50 cd	36.97 ± 0.91 g	12.9 ± 0.17 gh
	5	8.500 ± 0.77 ij	86.50 ± 1.53 gh	11.5 ± 0.02 bc	40.63 ± 0.29 e	14.5 ± 0.03 def
	10	10.17 ± 0.33 efgh	81.83 ± 0.33 i	10.2 ± 0.17 d	29.77 ± 0.07 i	12.2 ± 0.21 i
20 mW	0.5	10.50 ± 0.77 efg	85.17 ± 0.33 h	9.67 ± 0.17 de	40.14 ± 0.33 e	14.7 ± 0.25 cde
	1	10.83 ± 0.45 ef	89.17 ± 2.35 fg	11.3 ± 0.33 b	43.02 ± 0.27 cd	14.6 ± 0.24 cde
	3	10.33 ± 0.67 efgh	104.8 ± 1.92 b	12.5 ± 0.29 a	44.99 ± 0.61 ab	15.2 ± 0.11 c
	5	8.500 ± 0.02 ij	117.2 ± 0.60 a	13.0 ± 0.02 a	46.13 ± 0.28 a	18.9 ± 0.06 a
	10	8.167 ± 0.33 ij	93.67 ± 1.42 de	10.3 ± 0.17 ef	38.52 ± 0.16 f.	14.0 ± 0.18 ef
50 mW	0.5	8.167 ± 0.17 ij	90.83 ± 0.44 ef	9.00 ± 0.02 d	38.42 ± 0.43 f.	13.8 ± 0.36 f.
	1	9.500 ± 0.02 efgh	93.33 ± 0.89 de	9.83 ± 0.33 de	42.26 ± 0.50 d	14.2 ± 0.27 ef
	3	8.000 ± 0.04 j	95.50 ± 1.04 cd	10.3 ± 0.33 d	43.70 ± 0.47 bc	16.2 ± 0.13 b
	5	9.167 ± 0.17 ghij	92.67 ± 0.44 de	10.0 ± 0.29 d	38.55 ± 0.32 f.	14.9 ± 0.05 cd
	10	10.17 ± 0.33 efgh	88.67 ± 0.44 fg	9.00 ± 0.02 ef	36.42 ± 0.21 g	13.1 ± 0.08 g

Averages (means) in each column with the same letter (s) are not significantly different according to Steel et al., (1997) test with Bonferroni correction (*p* ≤ 0.05).

### Days of sprouting

In the present work, investigations days done to study the influence of He–Ne laser irradiation on the number of the days required for sprouting, vegetative growthparameters, flowering parameters, chemical composition, leaves anatomy, genetic attributes. According to data presented in Table 2, it is noticed that the three laser power levels (5, 20, 50 mW) at the tested exposure times (0.5, 1, 3, 5 and 10 min.) significantly decrease the needed period for sprouts appearance compared to control group. In both seasons, early sprouting was detected using laser irradiation, while a long period for corm sprouting was observed in control plants (19 days).

The minimum number of days (8–9 days) needed for corms sprouting was observed in plants treated with 5mW at irradiation time (3, 5 min), 20 mW at irradiation time (5,10 min) and 50 mW at all time exposure evaluated except 10 min irradiation. All laser treatments significantly enhanced early appearance of sprouts compared with control, Table 2.

### Flowering characteristics

The results recorded in Tables 3 and 4 indicated that, He–Ne laser radiation treatments affected significantly on a flowering date, the control plants gained the highest value of days of flowering (≈102 days), Meanwhile, the lowest value of early flowering was recorded with 20 mW for 5 min (67 days) as mentioned in Table 3. Additional data shows that laser treatments increased inflorescence characteristics**.** The vas life, flower FW (g/ plant), flower DW (g/ plant), No. flowers/inflorescence, flower diameter (cm), and inflorescence length (cm) were significantly affected (Table 3 and 4), the maximum increase was achieved at 20 mW for 5 min irradiation compared with the control.

**Table 3:** Flowering growth characteristics of (*Gladiolus grandiflorus* L.) cv.”White Prosperity” affected by the interactions between He–Ne Laser mW and different irradiation time during mean of seasons 2022/ 2023.

He–Ne laser (L)	Irradiation time (min)	Diameter of middle part of the plant (mm)	Leaf area (cm2)	Days to flowering	Vase life (days)
Control	0.5	43.260 ± 0.527 ij	55.007 ± 0.713 m	102.00 ± 0.289 a	7.500 ± 0.289 i
	1	43.797 ± 0.920 ij	56.133 ± 0.411 m	101.00 ± 0.289 a	7.667 ± 0.441 i
	3	42.688 ± 0.878 j	56.700 ± 0.759 m	101.17 ± 0.882 a	8.167 ± 0.333 hi
	5	42.687 ± 0.419 j	56.567 ± 0.188 m	100.83 ± 1.302 a	8.000 ± 0.289 hi
	10	44.713 ± 0.262 i	58.800 ± 0.464 l	101.67 ± 0.727 a	8.500 ± 0.289 ghi
5 mW	0.5	47.683 ± 0.073 h	71.257 ± 1.295 k	87.333 ± 0.441 bc	9.333 ± 0.441 efg
	1	48.978 ± 0.090 gh	86.413 ± 1.461 g	84.833 ± 0.833 cd	9.833 ± 0.601 def
	3	52.758 ± 0.535 cde	97.927 ± 0.678 cd	82.000 ± 0.500 e	12.00 ± 0.289 ab
	5	50.167 ± 1.126 fg	99.743 ± 0.348 c	84.500 ± 0.500 de	11.17 ± 0.441 bc
	10	51.610 ± 0.451 ef	90.703 ± 0.264 e	85.333 ± 0.333 cd	10.83 ± 0.167 cd
20 mW	0.5	52.110 ± 0.395 e	96.987 ± 0.686 d	85.833 ± 1.364 cd	10.33 ± 0.167 cde
	1	53.810 ± 0.362 bcd	98.678 ± 0.342 cd	89.833 ± 0.726 b	10.50 ± 0.289 cd
	3	55.032 ± 0.158 b	102.800 ± 0.35 b	90.000 ± 1.443 b	11.17 ± 0.726 bc
	5	57.283 ± 0.220 a	107.450 ± 0.40 a	67.500 ± 0.289 g	13.00 ± 0.577 a
	10	52.295 ± 0.069 de	88.638 ± 0.665 f.	83.667 ± 0.601 de	10.17 ± 0.167 cde
50 mW	0.5	54.718 ± 0.491 b	83.335 ± 0.698 h	84.000 ± 2.517 de	10.00 ± 0.500 de
	1	54.283 ± 0.753 bc	81.830 ± 0.703 h	88.833 ± 0.601 b	9.833 ± 0.167 def
	3	58.272 ± 0.776 a	87.645 ± 1.162 fg	75.167 ± 0.167 f.	10.17 ± 0.167 cde
	5	54.028 ± 0.400 bc	77.530 ± 0.209 i	87.500 ± 1.041 bc	8.833 ± 0.167 fgh
	10	52.745 ± 0.291 cde	74.222 ± 0.528 j	87.500 ± 0.289 bc	8.333 ± 0.167 ghi

Averages (means) in each column with the same letter (s) are not significantly different according to Steel et al., (1997) test with Bonferroni correction (*p* ≤ 0.05).

**Table 4:** Flowering growth characteristics of (*Gladiolus grandiflorus* L.) cv.”White Prosperity” affected by the interactions between He–Ne Laser mW and different irradiation time during mean of seasons 2022/ 2023.

He–Ne laser (L)	Irradiation time (min)	FW of flower (g/ plant)	DW of flower (g/plant)	No. flowers/inflorescence	flowers diameter(cm)	inflorescence length (cm)
Control	0.5	41.69 ± 0.49 opq	11.53 ± 0.063 k	8.33 ± 0.17 ghij	7.33 ± 0.44 gh	23.33 ± 1.09 g
	1	42.21 ± 0.19 nopq	12.78 ± 0.163jk	7.83 ± 0.17 j	6.83 ± 0.17 h	24.67 ± 0.17 g
	3	40.57 ± 0.38 q	13.59 ± 0.07 ij	8.17 ± 0.44 hij	8.00 ± 0.76 gh	22.83 ± 1.83 g
	5	42.93 ± 0.97 nop	14.25 ± 0.21 hi	8.17 ± 0.17 hij	7.17 ± 0.33 gh	25.67 ± 0.67 g
5 mW	10	43.87 ± 1.10 n	14.07 ± 0.81 hij	8.00 ± 0.29 ij	7.83 ± 0.60 gh	25.00 ± 0.76 fg
	0.5	57.37 ± 0.17 m	12.85 ± 0.56 j	6.83 ± 0.60 k	8.17 ± 0.67 g	29.00 ± 0.58 f.
	1	64.23 ± 0.89 hi	14.52 ± 0.61 ghi	8.83 ± 0.44 fgh	10.50 ± 0.50 ef	39.00 ± 0.57 e
	3	73.57 ± 0.81 c	18.21 ± 0.19 bc	10.83 ± 0.17 bc	11.50 ± 0.17 bcde	46.50 ± 0.50 cd
	5	70.70 ± 0.62 d	17.53 ± 0.38 bcd	9.30 ± 0.17 ef	10.83 ± 0.17 cdef	45.00 ± 1.15 d
20 mW	10	68.29 ± 0.31 ef	16.94 ± 0.65 cde	8.67 ± 0.17 fghi	10.00 ± 0.29 f.	44.83 ± 0.44 d
	0.5	59.48 ± 0.69 l	15.58 ± 0.13 fg	8.67 ± 0.17 fghi	10.67 ± 0.33 def	44.00 ± 0.57 d
	1	70.12 ± 1.12 de	15.74 ± 0.29gh	9.83 ± 0.17 de	10.67 ± 0.44 def	47.00 ± 0.57 cd
	3	84.55 ± 0.38 b	18.32 ± 0.97 b	10.33 ± 0.17 cd	10.67 ± 0.33 def	49.17 ± 0.61 bc
	5	89.00 ± 0.59 a	21.47 ± 0.18 a	13.00 ± 0.02 a	15.17 ± 0.60 a	54.00 ± 0.29 a
50 mW	10	66.96 ± 0.91 fg	17.07 ± 0.31 bcd	9.31 ± 0.17 ef	11.83 ± 0.17 bcd	49.67 ± 0.61 bc
	0.5	62.06 ± 1.28 jk	15.61 ± 0.61 fg	9.00 ± 0.00 fg	11.67 ± 0.67 bcde	46.83 ± 0.73
	1	63.38 ± 0.38 ij	15.05 ± 0.48 gh	9.83 ± 0.17 de	12.17 ± 0.33 b	45.50 ± 0.00 d
	3	65.48 ± 0.35 gh	20.50 ± 0.13 a	11.50 ± 0.00 b	14.00 ± 0.57 a	52.17 ± 0.17 ab
	5	60.38 ± 0.80 kl	16.53 ± 0.29 def	10.33 ± 0.17 cd	12.00 ± 0.29 bc	46.50 ± 0.77 cd
	10	56.70 ± 0.262 m	14.74 ± 0.40 ghi	9.83 ± 0.17 de	11.83 ± 0.33 bcd	46.67 ± 0.62 cd

Averages (means) in each column with the same letter (s) are not significantly different according to Steel et al., (1997) test with Bonferroni correction (p ≤ 0.05).

### Changes in (*Gladiolus grandiflorus* L.) cv.”White Prosperity” the color and shape of flowers after irradiated with different treatments of He–Ne laser radiation

**Figure 1:**
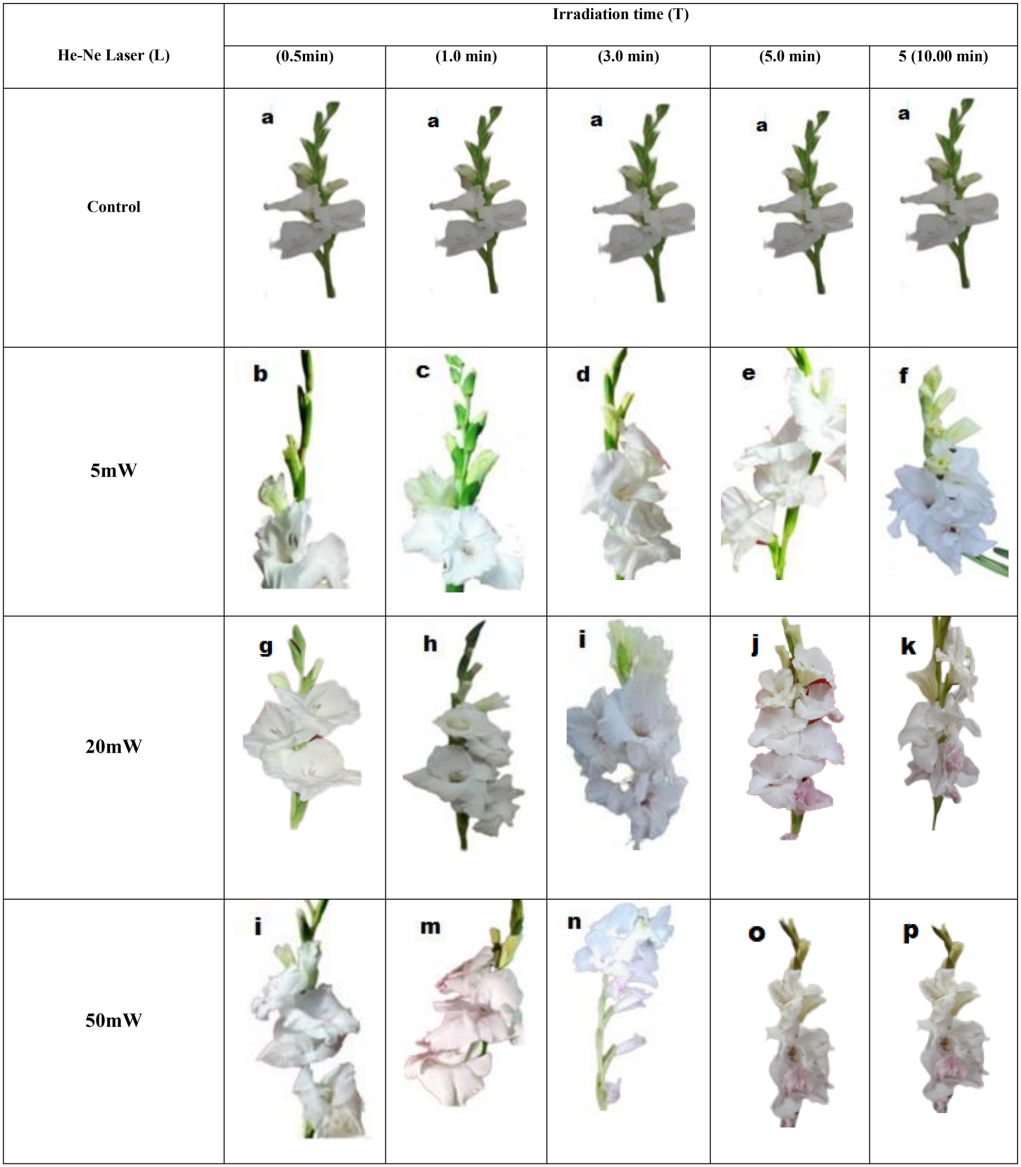
Illustrate Changes in (*Gladiolus grandiflorus* L.) cv.”White Prosperity” the color and shape of flowers after irradiated with different treatments of He–Ne laser radiation exposure times (0.0, 0.5, 1, 3, 5 and 10 min.) and power levels (5, 20, 50 mW).

The changes in the range of flower color and its shape were illustrated as shown in Fig. 1 as follows. The flowers of unirradiated plants were characterized by the clear white color of all the petals, white or yellow color. Treated plants with irradiation low laser irradiation (5 mW, 5 min) (Fig. 1b–e), normal flowers were obtained, while irradiation for 10 min resulted in change petals color to dark yellow (Fig. 1f). The irradiation with 20 mW for 0.5 min and 1.0 min. flowers will not be affected by radiation (Fig. 1g–l). Otherwise, irradiated plants either with He–Ne or with increasing irradiation time, clear difference was observed in inflorescences colors and growth. In addition, caused a change in color and petals compressed (Fig. 1), on the other hand dark red edges were observed, but this treatment gave the best flowers in shape, color, and quality (Fig. 1k). Increasing doses of irradiation to 10 min distorted the shape of the flower (Fig. 1l). However, it was noted that irradiation with a high dose at all levels led to a distortion of the general shape of gladiolus flowers, especially with an increase of 5 and 10 min.

### Corm characteristics and productivity

**Figure 2:**
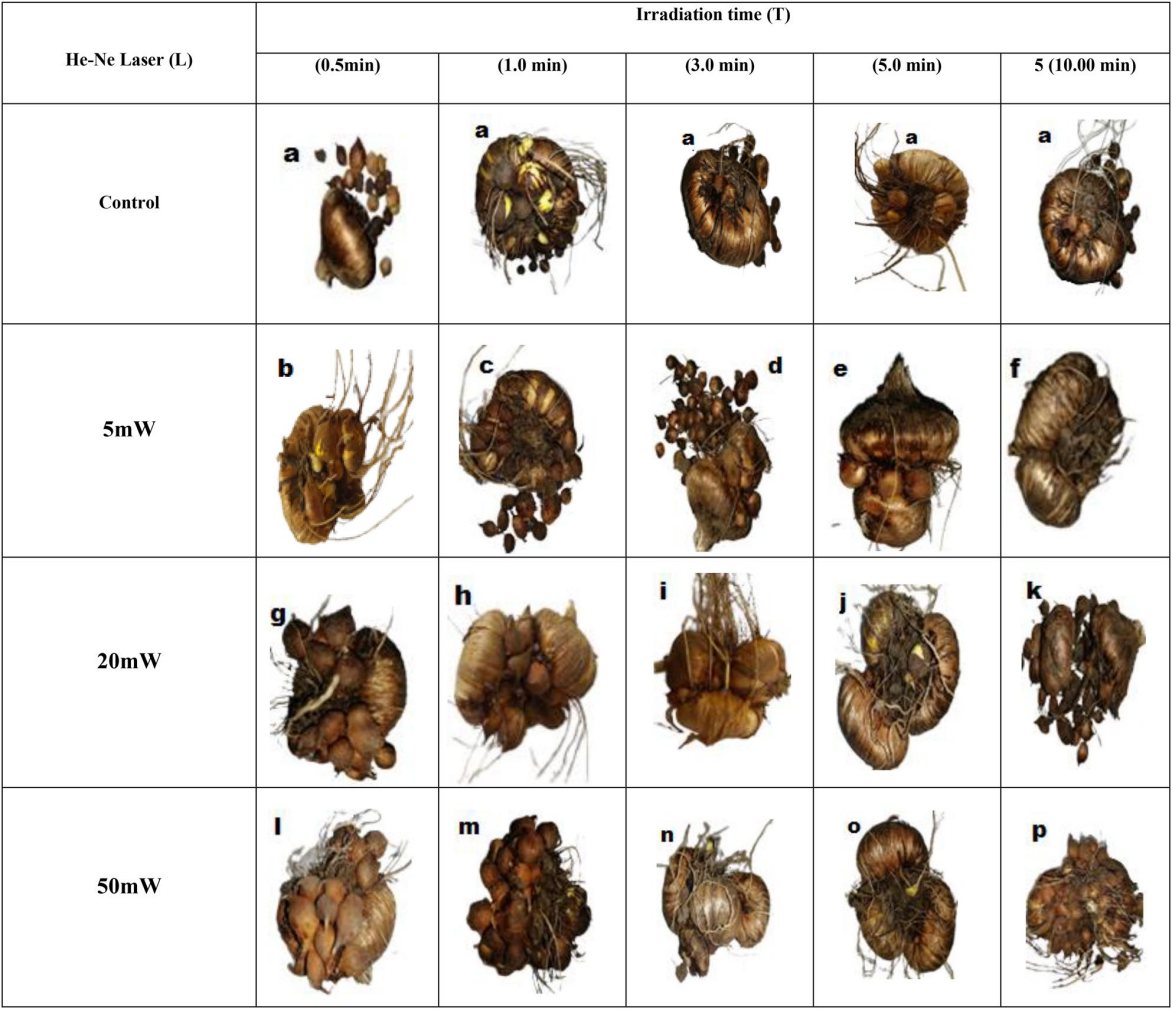
Changes in (*Gladiolus grandiflorus* L.) cv.”White Prosperity corms and cormlets morphology after irradiated with different treatments of He–Ne laser radiation exposure times (0.0, 0.5, 1, 3, 5 and 10 min.) and power levels (5, 20, 50 mW).

Corms are thick fleshy shortened stems, with a storage function analogous to the leaf scales of a bulb. After flowering, the base of the flower stem forms a new corm. The results recorded in Table 5 and Fig. 2, indicated that He–Ne irradiation treatments significantly affected the No. of corms/plant, FW of corms (g/plant), and circumference/corms. The medium level (20 mW) of He–Ne laser at 5 min. irradiation time gave the best values of the N. of corms /corm, FW of corms (g/plant), and hormones and enzymes like cytokinin and gibberellic acid are linked to plant growth (GA3).

**Table 5:** Corm characteristics of (*Gladiolus grandiflorus* L.) cv.”White Prosperity” affected by the interactions between He–Ne Laser mW and different irradiation time during mean of seasons 2022/ 2023.

He–Ne laser (L)	Irradiation time (min)	N. of corms/plant	FW of corms (g/plant)	Circumference/corms
Control	0.5	6.33 ± 0.17 i	25.33 ± 1.03 kl	5.90 ± 0.29 i
	1	6.83 ± 0.17 hi	25.75 ± 1.56 kl	6.10 ± 0.37 i
	3	7.00 ± 0.29 hi	26.73 ± 0.14 k	6.20 ± 0.18 i
	5	7.83 ± 0.44 h	25.52 ± 0.30 kl	6.28 ± 0.09 i
	10	7.00 ± 0.29 hi	23.80 ± 0.35 l	5.90 ± 0.39 i
5 mW	0.5	11.83 ± 0.44 fg	38.45 ± 0.20 e	8.02 ± 0.02 h
	1	14.17 ± 0.44 e	39.28 ± 0.03 e	8.43 ± 0.26 h
	3	20.33 ± 0.33 c	55.95 ± 0.47 c	12.7 ± 0.04 cd
	5	11.50 ± 0.29 g	32.87 ± 0.655 fg	12.0 ± 0.34 ef
	10	11.67 ± 0.44 fg	29.31 ± 1.11 ij	10.7 ± 0.05 g
20 mW	0.5	12.17 ± 0.44 fg	29.77 ± 0.11 hi	11.9 ± 0.47 ef
	1	19.00 ± 0.33 c	31.64 ± 0.83 gh	12.1 ± 0.02 def
	3	25.00 ± 0.33 b	34.54 ± 0.47f.	10.9 ± 0.07 g
	5	33.50 ± 0.75 a	61.14 ± 0.97 b	14.5 ± 0.22 a
	10	11.00 ± 0.33 g	27.34 ± 0.18 jk	12.8 ± 0.13 c
50 mW	0.5	12.33 ± 0.60 fg	29.03 ± 0.91 ij	12.5 ± 0.08 cde
	1	15.83 ± 1.17 d	33.23 ± 1.74 gh	12.3 ± 0.04 cdef
	3	19.50 ± 0.76 c	64.58 ± 0.34 a	13.5 ± 0.14 b
	5	13.00 ± 0.18 ef	42.98 ± 0.39 d	11.8 ± 0.17 f.
	10	13.00 ± 0.29 ef	37.44 ± 0.25 e	10.9 ± 0.14 g

Averages (means) in each column with the same letter (s) are not significantly different according to Steel et al., (1997) test with Bonferroni correction (*p* ≤ 0.05).

### Chemical composition

The illustrated results in Tables 6, 7 and 8 indicate that the highest values of total chlorophylls in leaves (SPAD), anthocyanin content (mg/100 g F.W) in petals, N%, P%, K%, and total carbohydrates in new corms (%), were recorded at 20 mW He–Ne irradiation at 5 min. On the other hand, the lowest values of total chlorophylls in leaves (SPAD), P%, K%, and total carbohydrates in new corms (%), were obtained from the control. By increasing laser dosage both power and irradiation time, high values of N%, anthocyanin content (mg/100 g F.W) in petals, total phenol (%) (μg CE/g) in corms, total flavonoid (%) (μg CE/g) in new corms, antioxidant (DPPH IC50 (μg/ml) (%) in new corms, and proline content in corms (μ moles/g) compared to the control.

**Table 6:** Mean square for the effect of He–Ne Laser (L), irradiation time (T) and their interaction on chemical composition of (*Gladiolus grandiflorus* L.) cv.”White Prosperity”.

Traits	Source of variation				
	Treatment			Error	Cv
	He–Ne Laser (L)	irradiation time (T)	L × T		
Total chlorophylls in leaves (SPAD)	1342.27**	112.339**	79.313**	0.673	1.612
Anthocyanin content (mg/100 g F.W) in petals	0.152N.S	0.004 N.S	0.001	0.000	4.324
N (%) in bulbs (%)	4.048*	0.117*	0.049*	0.066	11.89
P (%) in bulbs (%)	1.559**	0.081**	0.039*	0.002	0.530
K (%) in bulbs (%)	1.729***	0.260***	0.125***	0.001	1.410
Total carbohydrates in bulbs (%)	276.755**	28.628**	10.503**	0.261	1.551
Total phenol (μg CE/g) in bulbs (%)	18.386***	2.242***	0.554***	0.001	1.342
Total flavonoid (μg CE/g) in bulbs (%)	484.025***	18.889***	1.765***	0.077	1.625
Antioxidant (DPPH IC50 (μg/ml) in bulbs (%)	944.325***	79.600***	10.585***	0.185	1.323
Proline content in bulbs (μ moles/g)	202.329***	11.531***	2.624***	0.003	0.942

**Table 7:** Chemical composition of (*Gladiolus grandiflorus* L.) cv.”White Prosperity” affected by the interactions between He–Ne Laser mW and different irradiation time during mean of seasons 2022/ 2023.

He–Ne laser (L)	Irradiation time (min)	Total chlorophylls in leaves (SPAD)	Anthocyanin content (mg/100 g F.W) in petals		N (%) in bulbs (%)		P (%) in bulbs (%)		K (%) in bulbs (%)	
Control	0.5	35.51 ± 0.307q	0.102	± 0.002 g	1.380	± 0.006d	1.940	± 0.012 l	2.183	± 0.003 m
	1	36.24 ± 0.011opq	0.104	± 0.002 g	1.413	± 0.007d	1.963	± 0.007 k	2.220	± 0.006 m
	3	36.94 ± 0.194nop	0.103	± 0.001 g	1.443	± 0.015d	1.963	± 0.009 k	2.223	± 0.015 m
	5	38.21 ± 0.049 mn	0.104	± 0.001 g	1.420	± 0.006d	1.960	± 0.006kl	2.230	± 0.006 m
	10	39.54 ± 0.002 m	0.106	± 0.006 g	1.460	± 0.006d	1.943	± 0.012kl	2.330	± 0.085 l
5 mW	0.5	48.31 ± 0.127 k	0.188	± 0.008f.	2.137	± 0.003c	2.260	± 0.006j	2.347	± 0.009 l
	1	49.78 ± 0.098j	0.192	± 0.004f.	2.153	± 0.007c	2.283	± 0.003i	2.420	± 0.006 k
	3	53.18 ± 0.038hi	0.196	± 0.002f.	2.227	± 0.003c	2.320	± 0.006 h	2.460	± 0.012 jk
	5	58.33 ± 0.019 cd	0.199	± 0.006f.	2.277	± 0.003c	2.747	± 0.003 b	2.567	± 0.007gh
	10	55.85 ± 0.350ef	0.201	± 0.002f.	2.270	± 0.006c	2.613	± 0.003 d	2.487	± 0.003ij
20 mW	0.5	55.20 ± 0.009 fg	0.222	± 0.001e	2.240	± 0.012c	2.677	± 0.009c	2.540	± 0.012hi
	1	54.70 ± 0.328 fg	0.227	± 0.001e	2.327	± 0.009b	2.680	± 0.006c	2.587	± 0.003fgh
	3	57.23 ± 0.954de	0.277	± 0.001d	2.380	± 0.006b	2.727	± 0.012 b	2.637	± 0.015f.
	5	69.32 ± 0.003a	0.292	± 0.003 cd	2.540	± 0.015b	2.790	± 0.006a	2.853	± 0.007d
	10	59.42 ± 0.137 c	0.303	± 0.001c	2.467	± 0.009b	2.567	± 0.007e	2.747	± 0.015e
50 mW	0.5	50.80 ± 0.668j	0.299	± 0.008c	2.510	± 0.006b	2.473	± 0.007 g	2.613	± 0.007 fg
	1	54.31 ± 0.053gh	0.332	± 0.001b	2.490	± 0.031b	2.480	± 0.006 g	2.633	± 0.003f.
	3	66.87 ± 0.945b	0.336	± 0.001b	2.540	± 0.015b	2.670	± 0.006 c	3.650	± 0.006a
	5	52.38 ± 0.171i	0.370	± 0.025a	2.470	± 0.015b	2.687	± 0.003 c	3.193	± 0.044b
	10	45.83 ± 1.448 l	0.366	± 0.001a	3.087	± 0.662 a	2.537	± 0.009 f.	3.080	± 0.006c

Averages (means) in each column with the same letter (s) are not significantly different according to Steel et al., (1997) test with Bonferroni correction (*p* ≤ 0.05).

**Table 8:** Chemical composition of (*Gladiolus grandiflorus* L.) cv.”White Prosperity” affected by the interactions between He–Ne Laser mW and different irradiation time during mean of seasons 2022/ 2023.

He–Ne Laser (L)	Irradiation time (min)	Total carbohydrates in bulbs (%)		Total phenol (μg CE/g) in bulbs (%)		Total flavonoid (μg CE/g) in bulbs (%)		Antioxidant (DPPH IC50 (μg/ml) in bulbs (%)		Proline content in bulbs (μ moles /g)	
Control	0.5	25.61	± 0.307 l	1.42	± 0.009 m	10.21	± 0.004 k	22.13	± 0.016 m	2.13	± 0.009q
	1	26.67	± 0.272jk	1.47	± 0.010 lm	10.32	± 0.006 k	22.13	± 0.012 m	2.17	± 0.010q
	3	27.08	± 0.333j	1.48	± 0.007 l	10.65	± 0.282 k	22.27	± 0.047 m	2.19	± 0.019q
	5	28.15	± 0.032i	1.45	± 0.020 lm	11.24	± 0.010 j	22.33	± 0.013 m	2.21	± 0.007p
	10	26.00	± 0.321kl	1.49	± 0.009 l	11.25	± 0.006 j	22.34	± 0.016 m	2.24	± 0.006op
5 mW	0.5	32.17	± 0.537 g	1.63	± 0.017 k	11.98	± 0.348 i	28.14	± 0.030 l	2.37	± 0.115n
	1	30.95	± 0.410 h	1.68	± 0.003 k	12.71	± 0.175 h	29.44	± 0.020 k	2.65	± 0.015 m
	3	32.29	± 0.143 g	1.75	± 0.009j	13.40	± 0.087 g	29.02	± 0.289 k	3.16	± 0.012 l
	5	37.53	± 0.171b	1.88	± 0.003i	15.56	± 0.191 f.	32.17	± 0.042j	3.27	± 0.009 k
	10	35.22	± 0.094 d	1.95	± 0.017 h	15.96	± 0.020 f.	35.10	± 0.898 h	4.24	± 0.022j
20 mW	0.5	34.32	± 0.141e	1.85	± 0.005i	20.19	± 0.042 e	31.81	± 0.335j	4.88	± 0.006i
	1	32.82	± 0.333	1.97	± 0.003 h	20.46	± 0.100 e	33.25	± 0.101i	4.98	± 0.006 h
	3	36.47	± 0.110c	2.26	± 0.032 g	21.18	± 0.034 d	37.45	± 0.003f.	5.05	± 0.021 h
	5	39.03	± 0.007a	3.36	± 0.017e	21.44	± 0.029 d	39.23	± 0.109d	5.64	± 0.036 g
	10	33.14	± 0.012f.	3.55	± 0.070d	22.25	± 0.075 c	40.90	± 0.340c	7.30	± 0.053f.
50 mW	0.5	36.09	± 0.038c	2.98	± 0.003f.	20.31	± 0.032 e	36.42	± 0.088 g	7.85	± 0.037e
	1	36.83	± 0.304bc	3.35	± 0.015e	21.36	± 0.017 d	38.25	± 0.100e	9.22	± 0.007d
	3	39.13	± 0.010a	3.98	± 0.006c	22.18	± 0.035 c	39.36	± 0.074d	9.88	± 0.012c
	5	36.46	± 0.345c	4.43	± 0.012b	23.62	± 0.559 b	42.92	± 0.608b	11.44	± 0.016b
	10	32.82	± 0.664 fg	4.98	± 0.006a	25.47	± 0.056 a	45.68	± 0.067a	13.64	± 0.031a

Averages (means) in each column with the same letter (s) are not significantly different according to Steel et al., (1997) test with Bonferroni correction (p ≤ 0.05).

### Anatomical studies

**Figure 3:**
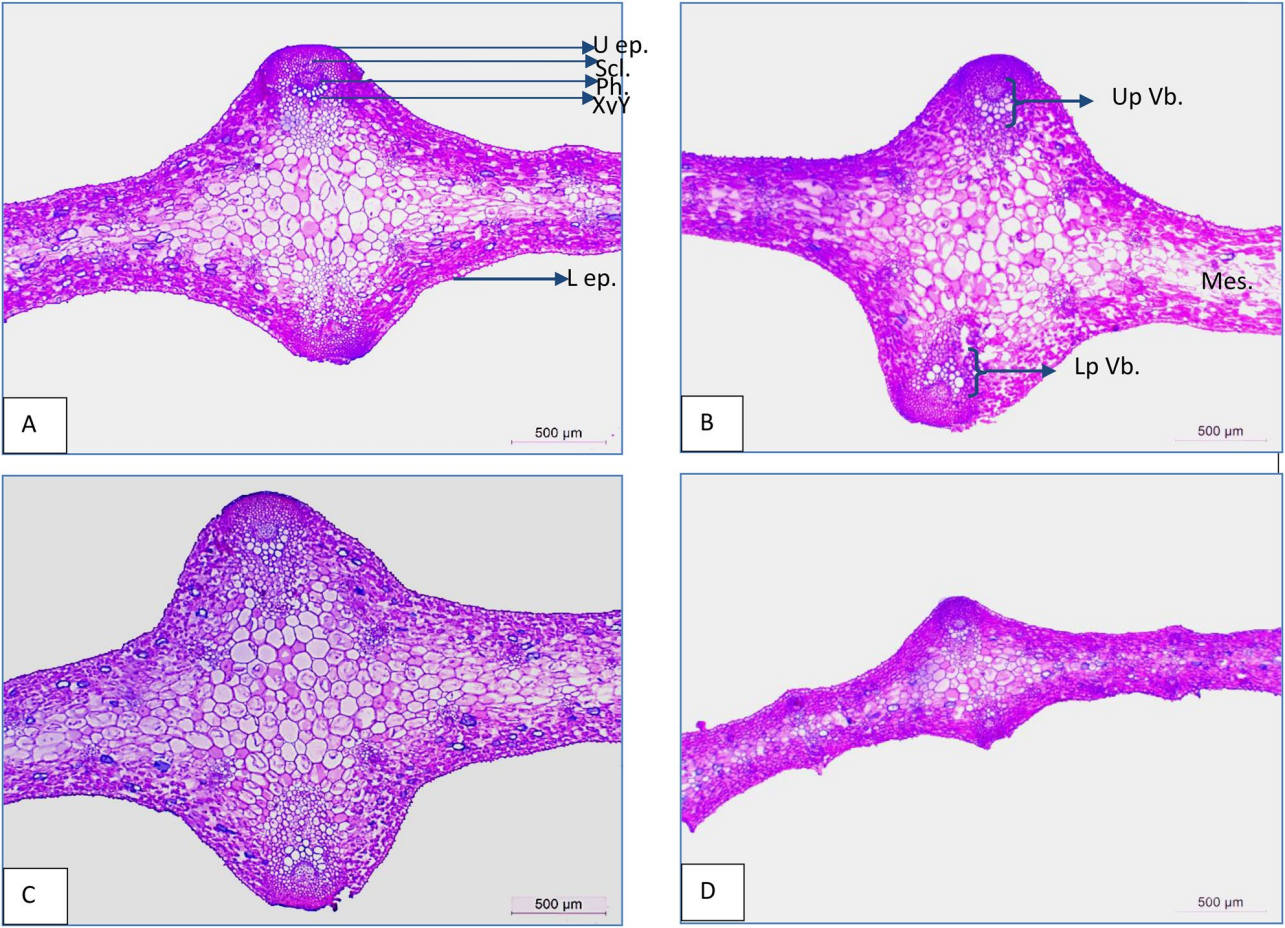
Anatomical changes in (*Gladiolus grandiflorus* L.) cv.”White Prosperity leaf after irradiated with different treatments of He–Ne laser radiation exposure time5min. and power levels (5, 20, and 50 mW. (A) Control B) plants irradiated with low power at 5mWfor 5min C) plants irradiated with medium power at 20mW for 5 min D) plants irradiated with50mW for 5 min. Details: U ep., Upper epidermis; Scl., Sclerenchyma tissue; Ph, Phloem tissue; Xy, Xylem tissue; L ep., Lower epidermis; Up Vb, Upper vascular bundle; Lp Vb, lower vascular bundle; Mes., Mesophyll tissue.

Transection made in untreated leaves of *Gladiolus grandiflorus* L. plants exhibited that the uniseriate layer with small papillae is present in both the upper (adaxial) and lower (abaxial) epidermal tissues. Adaxial epidermal cells are larger than abaxial epidermal cells. The veins have closed lateral vascular bundles in two rows and are distributed in two different sizes: large vascular bundles in the midvein region and small vascular bundles in the lamina region. The vascular bundles are surrounded by a single-layer sheath and each bundle has xylem towards the center of the leaf and phloem towards the surface of the leaf. There is a group of sclerenchyma cells above each bundle (phloem fiber cap). The mesophyll tissue appears to consist of many layers of spongy tissue. Moreover, tannin cells were clearly visible beneath the upper and lower lamina epidermal cells. The data indicated the effect of He–Ne laser dose power of 5 (low), 20 (medium) and 50 (high) mW for 5 min on the anatomical structure of gladiolus leaves as shown in Table 9 and in the cross-sections (Fig. 3). The results showed that the medium level (20 mW) of He–Ne laser at 5 min caused a vital increase in the leaf anatomical features, followed by the low level (5 mW) of He–Ne laser at 5min. An increment in midvein thickness was noted by such treatments. Compared with control plants, the percentages of increments in leaf midvein thickness were 29.83 and 19.59%, respectively.

**Table 9:** Anatomical changes in (*Gladiolus grandiflorus* L.) cv.”White Prosperity” leaves irradiated with different He–Ne laser power levels (5, 20, and 50 mW) and 5 min irradiation time.

Anatomical features (µ)	Control	5 mW He–Ne irradiation	20 mW He–Ne irradiation	50 mW He–Ne irradiation at 5 min
Upper epidermis thickness	24.37	24.48	30.36	18.03
Lamina thickness	616.30	590.21	781.52	254.68
Mesophyll tissue thickness	513.67	525.25	586.80	219.92
Midvein thickness	1789.63	2140.02	2323.51	552.89
Sclerenchymatous sheath thickness	132.84	146.26	172.87	68.47
midvein vascular bundle dimensions				
The upper bundle				
Length	229.74	256.24	289.44	93.90
Width	208.00	227.29	241.61	73.25
Phloem tissue thickness	63.70	76.06	87.12	26.48
Xylem tissue thickness	87.12	121.77	155.23	54.23
Xylem vessels diameter	25.79	33.75	47.73	23.09
The lower bundle				
Length	193.54	257.17	300.00	71.06
Width	228.22	200.96	248.02	51.08
Phloem tissue thickness	82.66	82.66	84.44	22.80
Xylem tissue thickness	121.69	129.24	131.55	40.85
Xylem vessel diameter	32.26	40.33	48.38	21.38
Sclerenchymatous sheath thickness	96.77	112.90	129.03	80.65
Lower epidermis thickness	17.67	22.46	28.84	12.81

### Molecular analysis

**Figure 4:**
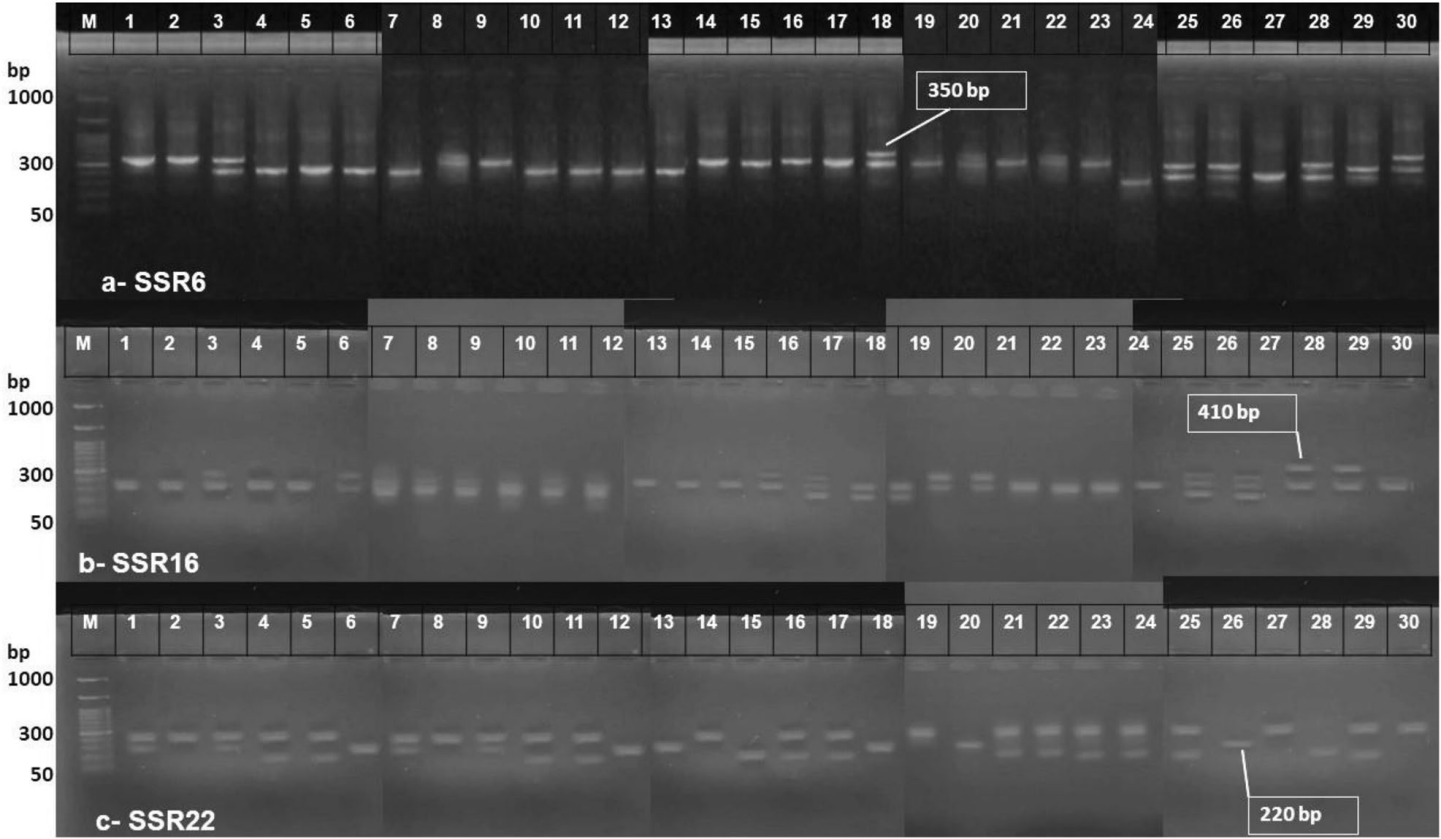
Patterns of SSR primers revealed by treated and non-treated Gladiolus against different LASER treatments. M = DNA ladder, [1] control 1 corm = CC1, [2] control 2 corm = CC2, [3] control Bulk 1 + 2 corm = BCC, [4] control 1 Flowers = CF1, [5] control 2 Flowers = CF2, [6] control Bulk 1 + 2 Flowers = BCF, [7] control 1 Leaves = CL1, [8] control 2 Leaves = CL2, [9] control Bulk 1 + 2 Leaves = BCL, [10] Control All Bulk = BC, [11] Medium 1 corm = MC1, [12] Medium 2 corm = MC2, [13] Medium Bulk 1 + 2 corm = BMC, [14Medium 1 Flowers = MF1, [15] Medium 2 Flowers = MF2, [16] Medium Bulk 1 + 2 Flowers = BMF, [17] Medium 1 Leaves = ML1, [18] Medium 2 Leaves = ML2, [19] Medium Bulk 1 + 2 Leaves = BML, [20] Medium All Bulk = BM, [21] High 1 corm = HC1, [22] High 2 corm = HC2, [23] High Bulk 1 + 2 corm = BHC, [24] High 1 Flowers = HF1, [25] High 2 Flowers = HF2, [26] High Bulk 1 + 2 Flowers = BHF, [27] High 1 Leaves = HL1, [28] High 2 Leaves = HL2, [29] High Bulk 1 + 2 Leaves = BHL, [30] High All Bulk = BH.

PCR products of 22 SSR primers were visualized on agarose gels and analyzed for variants induced in Gladiolus treated with different laser doses. The SSR primers exhibited various bands among the treated plants (Fig. 4). 112 bands emerged from the 22 SSR primers, ranging between 130 and 540 bp, with 32 bands having polymorphism ranging from 17 to 100% (Table 10). The highest band size was observed in primer SSR13 (540 bp), while the lowest was in primer SSR22 (130 bp). The average PIC was 0.63), MI was 1.61, and RP was 2.32 (Table 11). The maximum value of PIC (0.82) came from SSR2, whereas SSR22 revealed minimum PIC values of 0.28. The SSR15 recorded the greatest value of RP (3.01), whereas the lowest one by SSR4 (1.91). The MI was 2.32 as the greatest value recorded by SSR18, while the lowest one recorded by SSR22 (1.04) (Table 11).

**Table 10:** SSR marker parameters calculated across different LASER treatments applied to the Gladiolus.

Marker code	TF	MF	PF	P%	Allele size (bp)
SSR1	6	5	1	17	290–340
SSR2	5	4	1	20	230–270
SSR3	5	4	1	20	190–390
SSR4	4	4	0	0	240–365
SSR5	5	4	1	20	210–290
SSR6	6	0	6	100	220–365
SSR7	5	4	1	20	270–300
SSR8	4	3	1	25	220–290
SSR9	7	5	2	29	200–230
SSR10	5	4	1	20	210–280
SSR11	6	5	1	17	340–510
SSR12	5	5	0	0	500–540
SSR13	4	4	0	0	210–300
SSR14	5	4	1	20	170–290
SSR15	6	5	1	17	240–290
SSR16	8	0	8	100	243–386
SSR17	6	5	1	17	180–215
SSR18	6	6	0	0	160–300
SSR19	3	3	0	0	290–400
SSR20	4	4	0	0	280–400
SSR21	2	2	0	0	190–230
SSR22	5	0	5	100	130–405
Total	112	80	32		
Average	5.09	3.64	1.45

**Table 11:** SSR marker PIC, Rp and MI calculated across different LASER treatments applied to Gladiolus.

Marker code	Allele size (bp)	PIC	RP	MI	Marker code	Allele size (bp)	PIC	RP	MI
SSR1	290–340	0.59	2.3	1.9	SSR12	500–540	0.76	2.52	1.47
SSR2	230–270	0.82	2.69	1.9	SSR13	210–300	0.73	2.16	1.46
SSR3	190–390	0.73	2.01	1.45	SSR14	170–290	0.75	2.34	1.47
SSR4	240–365	0.59	1.91	1.91	SSR15	240–290	0.76	3.01	1.9
SSR5	210–290	0.77	2.57	1.43	SSR16	243–386	0.44	1.98	1.9
SSR6	220–365	0.74	2.3	1.47	SSR17	180–215	0.79	2.93	1.9
SSR7	270–300	0.77	2.58	1.48	SSR18	160–300	0.65	2.13	2.32
SSR8	220–290	0.29	1.94	1.47	SSR19	290–400	0.59	2.37	1.04
SSR9	200–230	0.77	2.54	1.48	SSR20	280–400	0.8	2.79	1.9
SSR10	210–280	0.55	2.04	1.9	SSR21	190–230	0.37	1.98	1.04
SSR11	340–510	0.41	1.98	1.48	SSR22	130–405	0.28	1.98	1.04
Average	PIC		RP		MI				
	0.63		2.32		1.61				

Out of the 22 SSR primers, 3 primers exhibited high polymorphism percentage, i.e., SSR6, SSR16 and SSR22 (Table 10). SSR6 exhibited 2 positive alleles which could be used as marker to differentiate the Gladiolus accessions. The first allele with 350 bp that was found in leaves generated from corms that were exposed to medium laser treatment, also found in Corms and their leaves which exposed to high treatment (Table 12). While the second with 290 bp which found in the flowers that produced from corms treated with high laser treatment. Four different alleles were observed in SSR16, the alleles with 144, 235 and 410 bp which found in leaves generated from corms that exposed to medium and high laser treatments; while allele with 131 bp observed in leaves that generated from corms exposed to medium treatment only. On the other hand, SSR22 exhibited the allele with 220 bp that appears with high treatment of corms and their leaves.

**Table 12:** Presence or absence of alleles among different primers used in Gladiolus.

SSR6								
Code	bp							
	309	350	315	290	250	240		
CC1	0	0	1	0	0	0		
CC2	0	0	1	0	0	0		
BCC	0	0	1	0	0	**1**		
CF1	0	0	0	0	1	0		
CF2	0	0	0	0	1	0		
BCF	0	0	0	0	1	0		
CL1	0	0	0	0	1	0		
CL2	0	0	1	0	0	0		
BCL	0	0	1	0	0	0		
BC	0	0	0	0	**1**	0		
MC1	0	0	0	0	**1**	0		
MC2	0	0	0	0	**1**	0		
BMC	0	0	0	0	**1**	0		
MF1	0	0	1	0	0	0		
MF2	0	0	1	0	0	0		
BMF	0	0	1	0	0	0		
ML1	0	0	1	0	0	0		
ML2	0	1	1	0	0	0		
BML	0	0	1	0	0	0		
BM	0	1	1	0	0	0		
HC1	0	0	1	0	0	0		
HC2	0	1	1	0	0	0		
BHC	0	0	1	0	0	0		
HF1	0	0	0	0	0	**1**		
HF2	0	0	0	1	1	0		
BHF	0	0	0	1	1	0		
HL1	0	0	0	0	1	0		
HL2	0	1	1	0	0	0		
BHL	0	1	1	0	0	0		
BH	1	1	0	0	0	0		

SSR16								
Code	bp							
	410	305	280	235	223	211	144	131
CC1	0	0	0	0	1	0	0	0
CC2	0	0	1	0	0	1	0	0
BCC	0	1	0	0	0	1	0	0
CF1	0	0	1	0	0	1	0	0
CF2	0	0	1	0	0	1	0	0
BCF	0	1	0	0	0	1	0	0
CL1	0	1	0	0	1	0	0	0
CL2	0	1	0	0	0	1	0	0
BCL	0	1	0	0	1	0	0	0
BC	0	0	0	1	0	0	0	0
MC1	0	1	0	0	0	1	0	0
MC2	0	0	0	0	0	1	0	0
BMC	0	0	1	0	0	1	0	0
MF1	0	0	0	0	1	0	0	0
MF2	0	0	1	0	0	1	0	0
BMF	0	1	0	0	0	1	0	0
ML1	0	1	0	**1**	0	0	0	1
ML2	0	0	0	**1**	0	0	0	1
BML	0	1	0	0	1	0	0	1
BM	0	1	0	0	1	0	0	0
HC1	0	1	0	0	0	1	0	0
HC2	0	0	0	0	0	1	0	0
BHC	0	0	1	0	0	1	0	0
HF1	0	0	1	0	0	1	0	0
HF2	0	0	1	0	0	1	0	0
BHF	0	1	0	0	0	1	0	1
HL1	0	1	0	0	1	0	1	0
HL2	1	1	0	**1**	0	0	0	0
BHL	1	1	0	0	1	0	0	0
BH	0	1	0	0	0	1	0	0

SSR22								
Code	bp							
	363	351	259	238	220			
CC1	0	1	**1**	0	0			
CC2	0	1	0	0	0			
BCC	1	1	**1**	0	0			
CF1	1	0	0	1	0			
CF2	1	0	0	1	0			
BCF	0	0	**1**	0	0			
CL1	0	**1**	0	0	0			
CL2	1	0	0	1	0			
BCL	1	0	0	1	0			
BC	0	0	**1**	0	0			
MC1	1	0	0	1	0			
MC2	1	0	0	1	0			
BMC	0	0	**1**	0	0			
MF1	0	**1**	0	0	0			
MF2	0	0	0	1	0			
BMF	1	0	0	1	0			
ML1	1	0	0	1	0			
ML2	0	0	**1**	0	0			
BML	0	**1**	0	0	0			
BM	0	0	0	1	0			
HC1	0	1	0	0	1			
HC2	1	0	0	1	0			
BHC	1	0	0	1	0			
HF1	1	0	0	1	0			
HF2	1	0	0	1	0			
BHF	0	0	**1**	0	0			
HL1	0	1	0	0	0			
HL2	0	0	0	1	0			
BHL	0	1	0	0	1			
BH	0	1	0	0	0			

[1] control 1 corm = CC1, [2] control 2 corm = CC2, [3] control Bulk 1 + 2 corm = BCC, [4] control 1 Flowers = CF1, [5] control 2 Flowers = CF2, [6] control Bulk 1 + 2 Flowers = BCF, [7] control 1 Leaves = CL1, [8] control 2 Leaves = CL2, [9] control Bulk 1 + 2 Leaves = BCL, [10] Control All Bulk = BC, [11] Medium 1 corm = MC1, [12] Medium 2 corm = MC2, [13] Medium Bulk 1 + 2 corm = BMC, [14Medium 1 Flowers = MF1, [15] Medium 2 Flowers = MF2, [16] Medium Bulk 1 + 2 Flowers = BMF, [17] Medium 1 Leaves = ML1, [18] Medium 2 Leaves = ML2, [19] Medium Bulk 1 + 2 Leaves = BML, [20] Medium All Bulk = BM, [21] High 1 corm = HC1, [22] High 2 corm = HC2, [23] High Bulk 1 + 2 corm = BHC, [24] High 1 Flowers = HF1, [25] High 2 Flowers = HF2, [26] High Bulk 1 + 2 Flowers = BHF, [27] High 1 Leaves = HL1, [28] High 2 Leaves = HL2, [29] High Bulk 1 + 2 Leaves = BHL, [30] High All Bulk = BH.

## Discussion

Data in Tables 1 and 2 showed that treated gladiolus corms with three laser power levels (5, 20, and 50 mW) resulted significantly increase in the studied growth parameters, (plant height, number of leaves/plant, leaf area, fresh and dry weight of leaves (g)/plant) compared with control. The obtained results were in agreement with other work done on mustard, cauliflower, and turnip plants, when they exposed to, He–Ne radiation, showed an increase in biomass, leaf count, and overall fresh weight^24^.

Similar outcomes were reported in the literature for medicinal sage^25^, *Curculigo orchioides*^24^, *Eustoma grandiflorum*^4^, *Adansonia digitata*^26^, and Ashwagandha^27^. The Red light accelerates the rhythm of plant growth producing an increase in root growth that increases plant height is another important indicator of plant growth. Red illumination also increases biomass and encourages plants' vertical development. As a result, comparable changes were seen in the leaf's length and width in accordance with this principle^28^. Authors asserted that cytokinin and GA, among other hormones and enzymes, engage in the growth, development, and reproduction of plants. The red light from laser irradiation is necessary for both the production and endogenous content of GA3 and GA146. The greater leaf area reflects laser beams effect which encourages cell division, raises GA levels, and subsequent growth during the vegetative stage^29^ and improved morphological and physiological traits^30^. As a result of the laser-mediated elevation of GA3, which was connected to various physiological processes including cell elongation, auxin, and sugar content; the plant's height, number of branches, and blooming stems were increased^31^. Plant growth and development, seed germination, and phytochrome function were all correlated with one another. The phytochrome system was stimulated by a red-light-emitting He–Ne laser, and responses were shown in lettuce^32^ at a wavelength of (632.8 nm).

In the present work, investigations have been done to study the influence of He–Ne laser irradiation on the number of the days required for sprouting, vegetative growthparameters, flowering parameters, chemical composition, leaves anatomy, genetic attributes. All laser treatments significantly enhanced early appearance of sprouts compared with control, Table 2.

The laser light mode of action has been mentioned in previous work^7^, laser light stimulant seed germination and plant development by turning light energy into chemical energy. Phytochromes that absorb laser light at a specific wavelength tend to boost the internal energy of seeds^33^. Our current findings are consistent with past findings of better germination in scorzonera^34^, and green beans seed^7^; all reported similar observations of improved germination ability with laser light treatment. A recent study by Khamis et al.^26^ found that *Adansonia digitata* seeds treated with a He–Ne laser had a higher percentage of seeds germinate than control seeds. On the contrary, Abou-Dahab et al.^4^ found that red laser radiation delayed bud flower initiation and the longest period was resulted from red light laser treatments in *Eustoma grandiflorum*.

He–Ne laser radiation treatments affected significantly on a flowering date. The results agree with Danaila et al.^35^ who reported that low irradiation time of He–Ne laser diode (660 nm, 200 mW) have significant positive differences in all studied and determined characteristics (growth rate, number of formed shoots, number of leaves formed, flower development in the two studied plants *Dianthus caryophyllus* and *Petunia hybrida*^35^. In addition, Abou-Dahab et al.^4^ worked on *Eustoma grandiflorum* most of the tested floral characters, days to flower bud initiation, days to bloom, Flowering percentage, No .of flower buds/plant, No. of flowers/plant, flower Diameter (cm), bloom stem length (cm.), Peduncle length (cm), days to flower senescence ( from blooming), No. of petals /flower, Petals area (cm^2^), No. of Stamens per flower, F.W. of flower (g), and D.W. of flower (g) were significantly increased when irradiated using red He–Ne at wavelength with irradiation output power 50 mW compared to control^4^. Enhanced results were recorded for the studied characters when the same author used blue light at 460 nm and green light at 530 nm.

Irradiated plants either with He–Ne or with increasing irradiation time, clear difference was observed in inflorescences colors and growth. In addition, caused a change on color and petals compressed (Fig. 1). From previous research, Abou-Dahab et al.^4^ changes in color and shape of *Eustoma grandiflorum* after times was reported. The range change of flower color and form were varied. as shown in (Fig. 1). He–Ne laser irradiation affected flowers to change into dark purple flowers for the exposure time of 20 min. While increasing time to 25 min flowers varied in the darkness of purple color from light to dark purple reddish and planed in white color.

He–Ne irradiation treatments significantly affected the No. of corms /plant, FW of corms (g/plant), and circumference/corms. The primary investigation found that the synthesis of GA3 and the endogenous GA1 content are significantly influenced by the red light of laser radiation. The GA3 response for cell elongation and other effects such as weakening the cell wall, production of proteolytic enzymes, increase of auxin content, increase of concentration of sugar, raising the osmotic pressure in cell soap^4^. The impact of laser light in the developmental processes is critical and it is necessary to understand the molecular mechanisms involved in various processes. This elongation of a cell that is treated with laser radiation led to an increase of plant height, number of branches, and number of flowers. Gibberellic acid (GA) and abscisic acid (ABA), the two main phytohormones, are known as endogenous regulators of seed germination, dormancy, plant growth, and development. In this research no investigations were done on plant hormones and enzymes but based on other work the effect of red laser increase plant height, number of branches, and number of flowers, which is directly affect corms morphology including No. of corms /plant, FW of corms (g/plant), and circumference of corms as found in the presented data^31^.

The results indicate that the total chlorophylls in leaves (SPAD), anthocyanin content in petals, N%, P%, K%, and total carbohydrates in new corms (%), were affected by He–Ne irradiation. Khamis et al.^26^ reported that the germination rate of *Adansoina digitata* was dramatically improved by low power laser therapy (10 mW/2 min). Additionally, the root and leaf lengths were evaluated in comparison to the control and another laser treatment at various powers and time intervals. Additionally, Marchant et al.^36^ indicated that the red photons in RB light appear to be the key to gene expression of *Curcuma longa* plants. Also, the red photons in RB light affect the synthesis of flavonoids and other antioxidants compared to the control and white illumination. In cucumber seedlings, the red light increased the net photosynthetic and chlorophyll content more than blue light^28^. Data in literature stated that utilizing laser energy raised the nitrogen level, which increased the protein content needed to develop plant organs such as the number of umbels and branches^37^. Laser light caused an increase in the number of cell membranes composed of phospholipids and nucleic acids as well as higher potassium and phosphorus concentrations that led to laser radiation-induced cell elongation. Other authors found the same results, laser irradiation involves raising nitrogen levels, which raise protein levels, growing plant organs including number of leaves and leaf area, number of branches, and umbels^37^. Responses of chlorophyll a, b, total chlorophyll and carotenoids to different He–Ne laser irradiation were analyzed A significant increase in total chlorophyll content was noted at 15 J/cm^2^ (*P* = 0.02) than control, among different pigments, the total chlorophyll showed a significant increment than control, whereas chlorophyll a, b and carotenoids show slight variations without significance as has been reported for cabbage and beet varieties^38^. Recently, Zielinska et al.^39^, have reported higher chlorophyll content in M. laevis with LED illumination than photosynthetic active radiation and different chlorophyll a/b ratio to light spectra and LED illumination based on the altered effect of cytokinin on photosynthesis. Similarly, improved chlorophyll synthesis was reported in Doritaenopsis^40^, with photosynthetic active radiation and tobacco with blue light. According to past studies, the pigment concentration in the leaves of the control and irradiation groups predicted the overall photosynthetic rate^41^. The current findings are consistent with He–Ne laser upregulation of genes associated to photosynthesis, including photosystem II PsbR. protein, ATP synthase, and chlorophyll a/b binding protein; and increased pigment content from laser radiation^42^. wheat treated to UV-B light showed improved electron transport chain efficiency of the ability to biosynthesis photosynthetic pigments^43,44^. Similar findings suggested that laser exposure could affect the chlorophyll biosynthetic gene, which is primarily responsible for protochlorophyllide synthesis^7,45^. Their investigation supported the findings that laser activation of photosystems I and II in brinjal plants might enhance pigment production and improve photosynthetic rate. Additionally, compared to the control group, the laser-irradiated groups showed a significant increase in stomatal conductance, transpiration rate, and concentrations.

The enhancement in leaf thickness due to laser treatments was associated with its role in improving GA formation and release of IAA which is reflected on cell elongation and growth and encouraged nutrient and water uptake^46^. Moreover, the increments in lamina thickness were linked with another increment in the thickness of the upper and lower epidermal tissues as well as mesophyll tissue thickness. Where the rates of increase in the previous three characteristics amounted to 24.58, 63.21, and 14.24%, respectively, when using the medium dose of laser radiation (20 mW) for 5 min. compared to the control. The largest vascular bundle, xylem, phloem tissue, and xylem vessels diameter were obtained after irradiation with medium power at 20 mW for 5 min. Compared with the control, the average increase percentages in the dimensions of upper and lower vascular bundles of the leaf midvein were 25.99 and16.16% for the upper bundle and 55.00 and 8.68% for the lower one in length and width, respectively. Furthermore, the percentage of increments in phloem, xylem tissue thickness, and xylem diameter in such bundles were 36.77, 78.18, and 85.07% in upper vascular bundle, 2.15, 8.10, and 49.96% in the lower one, more than control plants, respectively. Wider xylem vessels due to this treatment lead to increased water and nutrient uptake as well as plant growth. In this respect, Metwally et al.^47^ on *Celosia argentea* plants as well as Abu Dahab et al.^4^ on *Eustoma grandiflorum* plants found that laser irradiation caused an increase in vascular bundle dimensions (length and width) and lamina thickness. On the contrary, the higher level (50 mW) of He–Ne laser radiation resulted in a decrease in all anatomical features of leaves compared to control plants. From the anatomical results of gladiolus plants, it could be concluded that the positive effect of laser irradiation on plant growth may be due to its stimulation of the plant's ability to absorb light energy, which is transformed into energy stored in a chemical compound used in plant growth. In addition to its role in improving the potential energy of the plant hormone, which leads to the stimulation and activation of biochemical metabolism. It also improves mineral content, biomass, and antioxidant metabolism^48^. Laser irradiation that improves chlorophyll content, photosynthesis, and respiration in addition to increasing the activity of key enzymes contributes to the biosynthesis of phenylpropanoids and coumarins^49^.

The SSR primers exhibited various bands among the treated plants (Fig. 4). 112 bands emerged from the 22 SSR primers, ranging between 130 and 540 bp, with 32 bands having polymorphism ranging from 17–100% (Table 10). These finding revealed the efficiency of SSR primers for differentiating Gladiolus plants, our finding are in harmony with Hiremath et al.^50^ who used SSR in studying the Genetic diversity and structure analysis for efficient utilization and sustainable management of gladiolus germplasm. On the other hand, our finding revealed that some alleles were affected by laser in their corms and the gene expression was resulted color or abnormalities aspects in leaves and /or flowers. Mutation in some alleles could result abnormalities like mutation in the allele with 410 bp revealed by SSR16. Thess finding are in harmony with Shehata et al.^7^, who demonstrated that the maximum value of GTS was %40% at 20-milliwatt power for 120min, while the minimum value was recorded at 22.5%at5-milliwatt power for 30s and the control.

## Methods

### Field experiment and laser treatments

A field experiment was conducted at the Nursery of Ornamental Horticulture Department, Faculty of Agriculture, Cairo University, Egypt, during the seasons of 2021 and 2022 to study the response of growth, quality characteristics, genetic attributes, and anatomy of *Gladiolus grandiflorus* against several red laser light treatments. Corms of *Gladiolus grandiflorus* cv. “White Prosperity” were provided by blooms nurseries, Cairo, Egypt with 10–12 cm in circumference and 20–30 gm in weight. The corms of 2.8–3.1 cm in diameter were selected and cured at ambient temperature (28 ± 2 °C) till July and stored under low temperature (5 °C) for 2 months to break their dormancy. Corms were pre-illuminated using He–Ne continuous laser with a wavelength at 630 nm, as light source (equipment whitening, LASER II, DMC Equipment Ltd.). Corms were divided into five groups with five replications for each according to the exposure times (0, 0.5, 1, 3, 5 and 10 min), and power levels [5(low), 20 (medium), and 50 mW (high)], the unirradiated corms were used as control. Then corms planted by the 6th of October on rows in open field. Corms planted on one side of the row, at a spacing of 20 cm within the row, and at a depth of 8–10 cm. All practices, i.e., irrigation and fertilization, were applied as commonly recommended.

### Morphological measurements

The following morphological and chemical constituent measurements were conducted on the gladiolus plants:

Sprouting, days to corms sprouting.

Vegetative growth characteristics, Plant height (cm), number of leaves per plant, fresh and dry weights of leaves (g), plant diameter (mm), at 5 cm above soil surface, and leaf area (cm^2^).

Flowering characteristics, Days to flowering (days), number of flowers per inflorescence, flowers diameters (cm), at the third flowers per inflorescence from the bottom of each spike), inflorescence length (cm), flowers fresh and dry weights per inflorescence (g).

Vase life of flowering (days), After cut flowers were transferred to 500 ml glass jars containing 300 ml of Distilled water was used for the control and to prepare the tested solutions. 2% sucrose was added to all treatments including the control treatment for improving postharvest vase life and quality of flowering.

Corms characteristics, Number of corms per plant, fresh weight of corms and corms circumference.

### Chemical constituents

Photosynthetic pigments of leaves: Leaf chlorophyll using the SPAD-502 m (Konica-Minolta, Japan).

Total anthocyanins content of petals (mg/100g petal fresh weight) determined in the third flower from the bottom of each inflorescence was excised after harvest and the petals (100 mg) were diced^51^.

Total carbohydrates % in the newly formed corms (cormels) and leaves were determined in dried leaves samples^52^.

Proline content in dried corms (μ moles/g)^53^.

Total phenols and indole acetic acid^54^.

Minerals content N, P, and K were determined in corms. Nitrogen was determined by using Kjeldahl methods^55^, Phosphorus was determined by using spectrophotometer^56^, while potassium concentration by flam photometer apparatus (BWB-1).

### Anatomical structure

Specimens in the second growing season at the age of two months were collected from the medium parts of the leaves of the main stems, killed and fixed in F.A.A. buffer (10ml formalin, 5ml glacial acetic acid, and 85 ml ethyl alcohol 70%) for 48 h. then anatomical process was carried out according to^57^.

### Molecular genetic studies

#### DNA extraction, primers and SSR markers amplification

DNA separated from irradiated corms and their green leaves and petals as described by Khaled et al.^10^. 22 SSRs primers were constructed according to Hiremath et al.^50^. (Table 13). Amplification has proceeded as follows, 94 °C for 1 min (one cycle); 94 °C for 20 s, 50–55 °C for 35 s, 72 °C for 45 s (35 cycles) and the last extension at 72 °C for 45 s (one cycle). The PCR products and DNA Marker Ladder, 11 Fragments (50–1000 bp) were run on electrophoresis at 90 V, in 2% agarose gel containing 0.5 μg/ml ethidium bromide for 2 h. The gel was imagined under UV.

**Table 13:** Twenty-two microsatellite characterizations used in *Gladiolus grandiflorus* cv. “White Prosperity” investigation.

	Primers code	Forward Primer sequence (5′–3′)	Anneal. °C	Reverse Primer sequence (5′–3′)	Anneal. °C
1	SSR1	TGCCACTCCAGCATAACTTCTA	60	ACTCCTTTTCCTCCCATTCTTC	60
2	SSR2	GGCATCCTTCCTCTCCGT	60	CGGCCTTGGGTGTAGAAGTAG	60
3	SSR3	GGTATGGAAACCTGCTAAGTGG	60	TAGATCCACAATTTCTCCCCAT	60
4	SSR4	GGAGACTGACTAGGGCAAAAGA	60	AACTCCTTGACGCATTACGACT	60
5	SSR5	GAAGAAGCGTGAGAAATCCATA	58	GGACCAACCGCAATAAATAA	58
6	SSR6	TCTATGTCAGTGCTCTACCGGA	60	GAAGCAAACGAGTCTGTGGAC	60
7	SSR7	AAACCCTCACTTCGGAGATCA	54	TAAAGTCAGTCAGCTGTAACACTG	54
8	SSR8	TTGTTACTGGTGCGGACTCC	58	CAGGTCCGATTGCTTGAGGA	58
9	SSR9	CCAAGTAAGTGATGGCGGC	56	GGGTCTAGAGAAGGCTTGGG	56
10	SSR10	AGAGAAGAGAGCATGGCGATA	46	GCGAGAAGTGGCATAAAGAGA	46
11	SSR11	CCCTAGCAAACATCTCTTCCA	54	TGTTATCAGCAAGCAGTCCAG	54
12	SSR12	AAAGTCCCTCCTCTCCTCTGAT	60	GAGCTTGTTACTGAACGGAACC	60
13	SSR13	CCTTGGTATGGAAACCTGCTAA	60	ATAGATCCACAATTTCTCCCCA	60
14	SSR14	GGAAGCTGTTCTAACGAATGGA	61	TATTGGGGATAGAGGGACTTGA	60
15	SSR15	GCTCACAACAATAATCCTTCCC	60	CAATGAACTCAGCAATACCAGC	60
16	SSR16	GTGTCTTCGGTGCTTTTCTCTT	60	CAGCGATAACCTAGAACGAACA	59
17	SSR17	GGGTCATCGCCTGTCATGAA	54	TCGTATCGGCTTGTTGGCTG	54
18	SSR18	ATGCCTTTGTCCTCTCACCT	54	TTTGTCCCTAATTGGAACACGTC	54
19	SSR19	TCTCCTCCTGTCCGTCTATCC	45	AGTCGTCCAAATCTCCGAACT	45
20	SSR20	AAGCAAAAGGTTTTCCATTCC	52	GTTTCTTGTCGAGGAACATGC	52
21	SSR21	GGGTTTGTATTGTTTGTTGGAGA	60	GGGTGATGTGGTCCTTGTAGA	60
22	SSR22	TATAGAGGAATGCGTGTCCGAT	61	TACTGCATGACGAGGAAATCAC	60

### Statistical analysis and data scoring

This experiment was factorial [5 exposure time treatments × 4 irradiation treatments (including the control)] and conducted using a randomized complete design with three replicates for each treatment as well as the controls, and 20 corms were used in each treatment. The recorded data were subjected to one-way analysis of variance according to Snedecor and Cochran^58^. The values of the least significant difference and the means were compared using the least significant difference test at 5% levels of probability. The banding patterns generated by the SSR primers were scored as 0 and 1 of bands for absence and presence. The number of polymorphic and monomorphic bands recorded, as well as the total number of bands and polymorphism%, were all determined by the banding patterns observed and produced by SSR primers. Furthermore, the ability of markers to estimate genetic variability was evaluated by measuring resolving power (RP) from Gorji et al.^59^, effective multiplex ratio (EMR), marker index (MI) from Varshney et al.^60^, and polymorphism information content (PIC) from De-Riek et al.^61^.

### Ethical approval

The authors confirm that experimental research on the gladiolus plant (*Gladiolus grandiflorus* cv. “White Prosperity”), including the collection of plant material, complied with institutional, national, and international guidelines and legislation.

## Conclusions

In the current research, Corms of *Gladiolus grandiflorus* cv. “White Prosperity” was irradiated using He–Ne at 635 nm at various exposure times in minutes (0.0, 0.5, 1, 3, 5 and 10 min.), and three power levels, low, medium, and high in milliwatt (mW) (5, 20, 50 mW). Vegetative growth parameter including (spouting days, Plant height (cm), leaves number, fresh and dry weights (g/plant), plant diameter (mm) and leaf area (cm^2^), floral parameters including (flowering days, vase life/day), fresh and dry weights of flower (g/plant), number of flowers per spike, spike length(cm), flower diameter(cm), number of corms per corm, corms fresh weight(g/plant) Circumference/corms), pigments (Total chlorophylls in leaves (SPAD), Anthocyanin content (mg/100 g F.W) in petals, NPK (%) in pulp and chemical composition in bulbs total carbohydrates (%),total phenol (μg CE/g (%),total flavonoid (μg CE/g) (%),Antioxidant (DPPH IC50 (μg/ml (%), and Proline content (μ moles /g). All mentioned studied characters illustrated that the medium irradiation power (20 mW for 20 mW for 5 min irradiation significantly increased compared with the control group and other irradiation powers and times. The results showed that the medium level (20 mW) of He–Ne laser at 5 min caused a vital increase in the leaf anatomical features, followed by the low level (5 mW) of He–Ne laser at 5min. Wider xylem vessels due to this treatment lead to increased water and nutrient uptake as well as plant growth. Improving GA formation and release of IAA which is reflected on cell elongation and growth and encourages nutrient and water uptake. Reduction in some of the studied morphological characters were recorded by increasing laser power 50mW and exposure time and resulted in a decrease in all anatomical features of leaves compared to control plants. The author concluded that high laser power or long exposure time may negatively affect the plant growth and some of the studied parameters. Prospective laser studies may use lower power and irradiation time. 112 bands emerged from 22 SSR primers, ranging between 130–540 bp, with 32 bands having polymorphism ranging from 17–100%. Out of the 22 SSR primers, 3 primers exhibited a high polymorphism percentage, i.e., SSR6, SSR16 and SSR22 which exhibited 7 positive markers. These findings revealed the efficiency of SSR primers for differentiating Gladiolus plants and revealed that some alleles were affected by laser in their corms and the expression resulted in color or abnormalities in leaves and/or flowers. Mutation in some alleles could result in abnormalities like mutation in the allele with 410 bp revealed by SSR16 (Supplementary Figure 4).

### Supplementary Information

Supplementary Figure 4.

## Data Availability

The data that support the findings of this study are available from the corresponding author upon reasonable request.
