# Supplementary material for: Laser-induced changes in the gene expression, growth and development of Gladiolus grandiflorus cv. “White Prosperity”
Source: Sci Rep. 2024 Mar 15;14:6257. doi: 10.1038/s41598-024-56430-6 (PMC10943131; doi:10.1038/s41598-024-56430-6)
Supplement: Supplementary file 1 — Supplementary Figure 4. [file 41598_2024_56430_MOESM1_ESM.pdf]

Submission ID aa765bd7-750f-436f-a938-d9914e5b6f20

Laser-Induced Changes in the gene expression, **Growth and Development of *Gladiolus grandiflorus*** cv. "White Prosperity"

Manar Hassan[1], Shimaa A. Shaaban[2], Rasha A. El ziat[3] and Khaled A. Khaled[4]*

corresponding author: Khaled.adly@agr.bsu.edu.eg

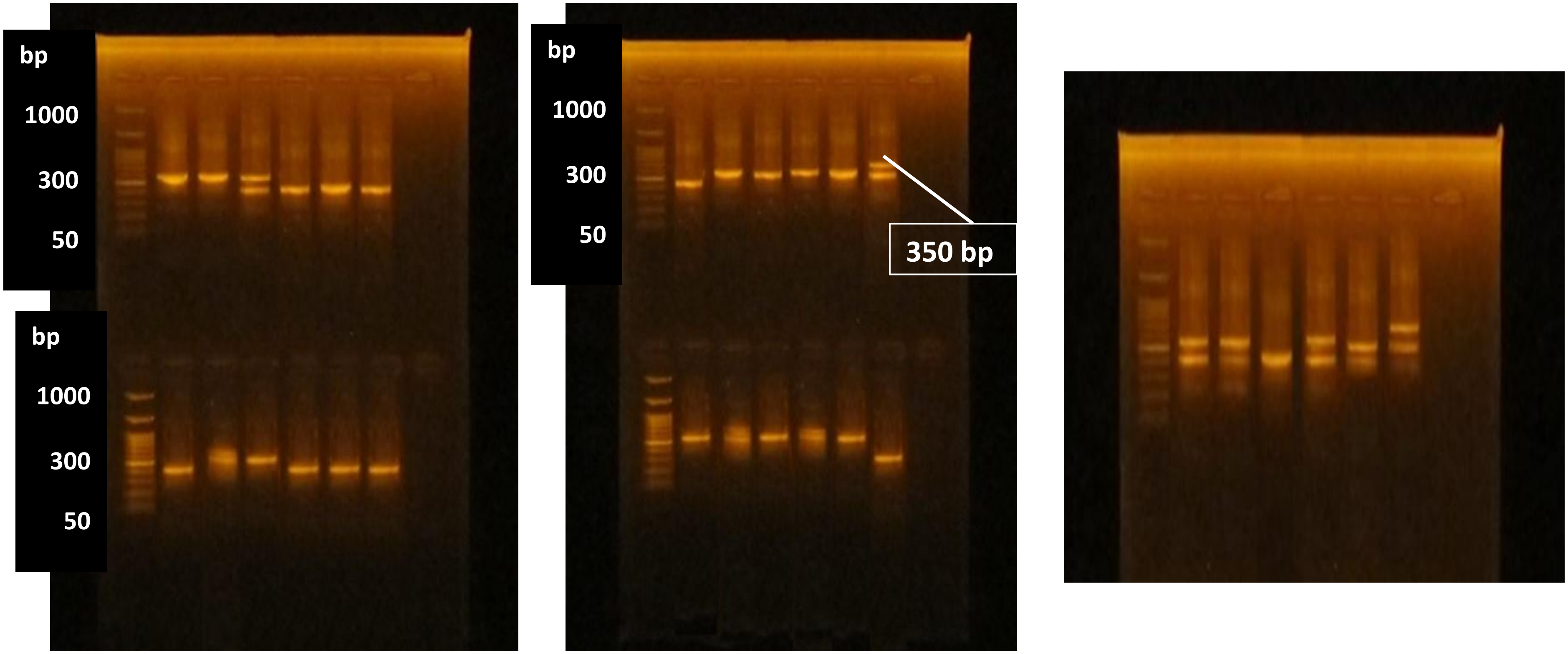

SSR6 (1-12) **SSR6 (13-24)** **SSR6 (25-30)**

Fig. (4a). Patterns of SSR primers revealed by treated and non-treated Gladiolus against different LASER treatments. SSR6

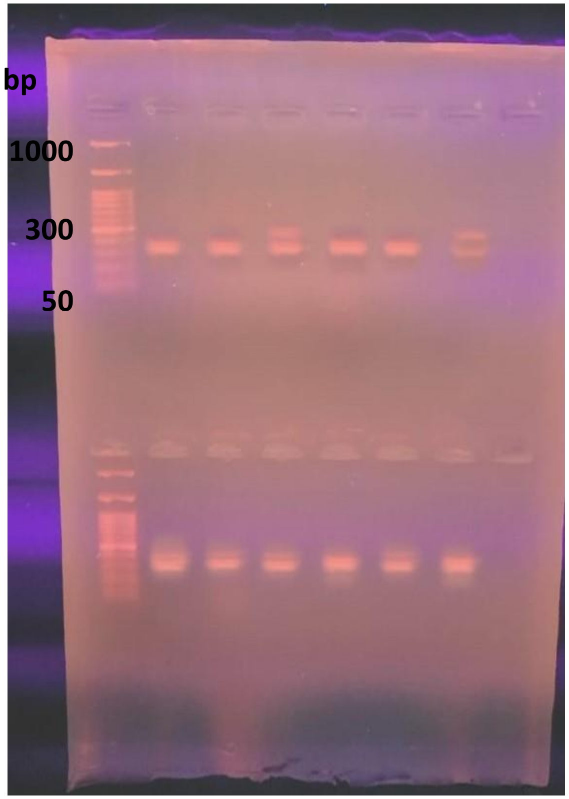

SSR16 (1-12)

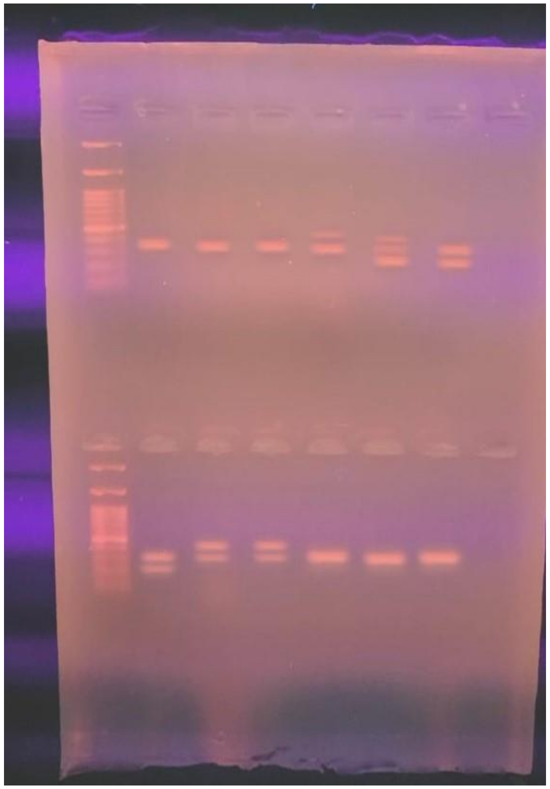

SSR16 (13-24)

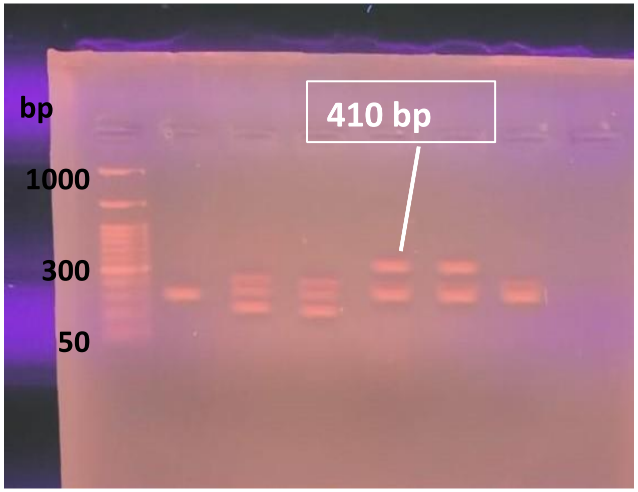

SSR16 (25-30)

Fig. (4b). Patterns of SSR primers revealed by treated and non-treated Gladiolus against different LASER treatments. SSR16

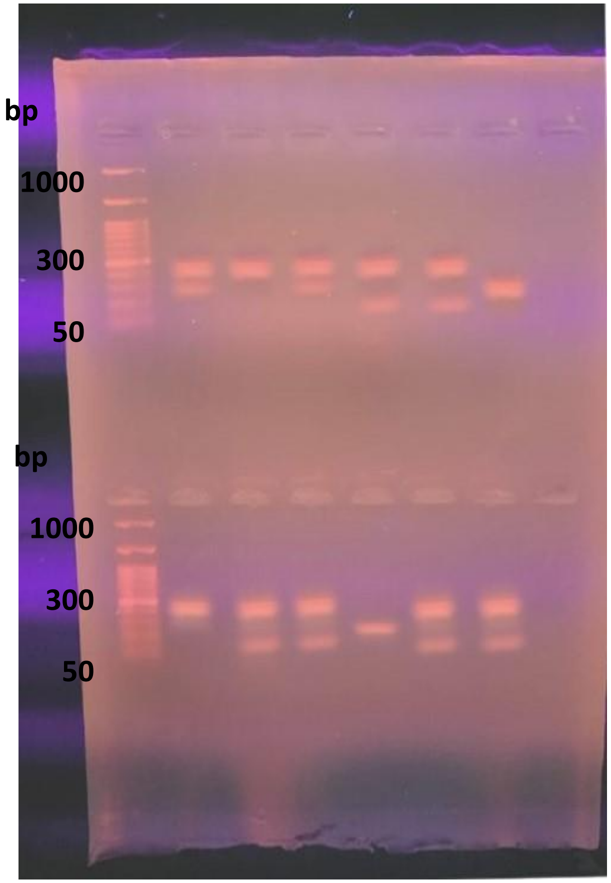

SSR22 (1-12)

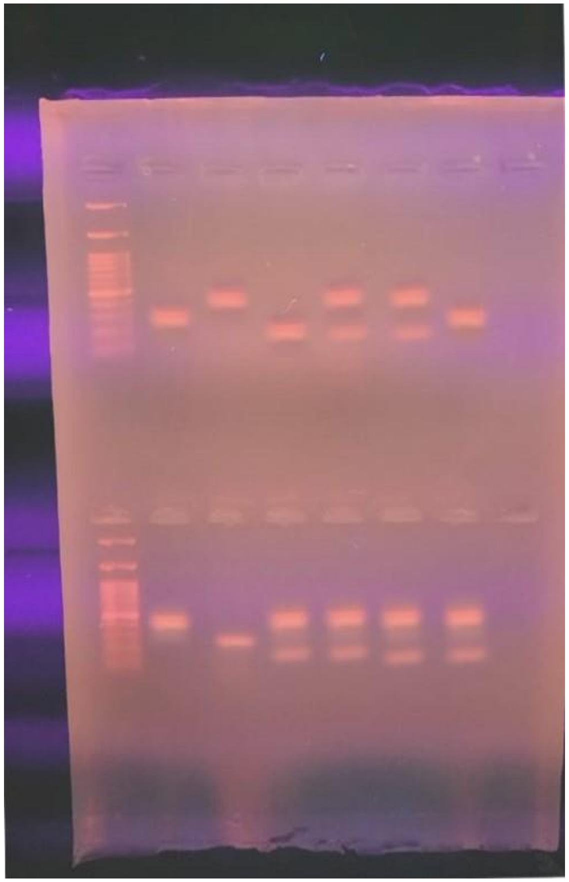

SSR22 (13-24)

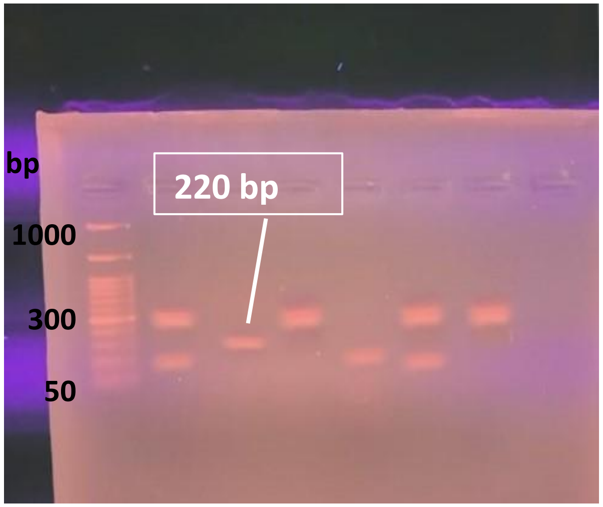

SSR22 (25-30)

Fig. (4c). Patterns of SSR primers revealed by treated and non-treated Gladiolus against different LASER treatments. SSR22
